# Towards accurate characterization of clonal heterogeneity based on structural variation

**DOI:** 10.1186/1471-2105-15-299

**Published:** 2014-09-08

**Authors:** Xian Fan, Wanding Zhou, Zechen Chong, Luay Nakhleh, Ken Chen

**Affiliations:** Department of Bioinformatics and Computational Biology, The University of Texas M.D. Anderson Cancer Center, 1515 Holcombe Blvd, Houston, TX 77030 USA; Department of Computer Science, Rice University, 6100 Main St, Houston, TX 77005 USA

**Keywords:** Structural variation, Clonal heterogeneity, Variant allele fraction

## Abstract

**Background:**

Recent advances in deep digital sequencing have unveiled an unprecedented degree of clonal heterogeneity within a single tumor DNA sample. Resolving such heterogeneity depends on accurate estimation of fractions of alleles that harbor somatic mutations. Unlike substitutions or small indels, structural variants such as deletions, duplications, inversions and translocations involve segments of DNAs and are potentially more accurate for allele fraction estimations. However, no systematic method exists that can support such analysis.

**Results:**

In this paper, we present a novel maximum-likelihood method that estimates allele fractions of structural variants integratively from various forms of alignment signals. We develop a tool, BreakDown, to estimate the allele fractions of most structural variants including medium size (from 1 kilobase to 1 megabase) deletions and duplications, and balanced inversions and translocations.

**Conclusions:**

Evaluation based on both simulated and real data indicates that our method systematically enables structural variants for clonal heterogeneity analysis and can greatly enhance the characterization of genomically instable tumors.

**Electronic supplementary material:**

The online version of this article (doi:10.1186/1471-2105-15-299) contains supplementary material, which is available to authorized users.

## Background

Tumor development is driven by oncogenic alleles created by somatic mutations [1]. These alleles are present in a tumor sample at different fractions, resulting from a history of clonal expansion [2]. Obtaining accurate estimation of variant allele fractions (VAFs) has become a critical step towards delineating the clonal architecture of a tumor sample and identifying driver mutations that promote clonal expansion [3–5].

Despite rapid progress in the next generation sequencing (NGS), challenges remain in accurately estimating VAFs. At a sequencing coverage of 50-100x, such as those obtained from standard whole-genome sequencing (WGS) or whole-exome sequencing, it is difficult to distinguish sub-clonal difference based on allele fractions estimated from single nucleotide variants (SNVs) and small indels [6]. At deeper (500-1000x) coverage, it is feasible to distinguish a few clones [3–5, 7]. Unfortunately, obtaining and analyzing such deep coverage at a whole-genome scale is still prohibitively expensive. Even at such high coverage, substantial variations in VAFs have been observed among variants from the same clone, making it challenging to infer the number of clones and assign variants to clones [8]. Megabase (Mb) long chromosomal aberrations have long been utilized in pathology laboratories to characterize heterogeneity in a tumor sample [9]. Recently, they have been utilized to perform early detection in circulating cell-free DNA samples [10]. Industrial scale application of paired-end short-insert NGS has made it possible to extend such characterization to smaller SVs that are of kilobase (Kb) in length [11]. Most NGS DNA libraries have higher physical coverage than sequence coverage, i.e., the DNA insert sizes are more than twice longer than the read lengths. This makes structural variants (SVs), including small (Kb) SVs and balanced SVs, more accurate targets than SNVs and indels for VAF estimation.

It is shown from WGS that there are typically tens to hundreds of somatic SVs in a genomically instable colorectal or breast cancer genome [12–14]. Potentially more exist at sub-clonal levels in a heterogeneous sample [15]. Therefore, enabling VAF estimation of SVs can substantially impact mutational profiling and tumor heterogeneity analysis.

Unfortunately, it is not straightforward to quantify the amount of evidences that is specifically associated with an SV allele from sequence alignments, which are usually obtained from aligning individual reads or read pairs to the human reference genome [16]. Depending on the type of SV and the results of alignment, signals that indicate the presence of an SV may appear in several different forms such as read depth, discordant read pairing, read split-alignment, end-clipping, unmapped read and so on [17]. Many tools only utilize one form of signal [18]. For example, THetA [19] and ABSOLUTE [7] utilize only read depth (or counts) from large (Megabase) copy number variants (CNVs). Thus, they cannot be applied to estimate the allele fraction of balanced SVs such as inversions and reciprocal translocations. They are also limited in measuring smaller SVs, which often occur more frequently than larger SVs. BreakDancer [20] utilizes only discordant read pairs, while CREST [21] and Pindel [22] utilize only split-reads. Several recent tools such as GenomeSTRiP [23], Delly [24] and ERDS [25] utilize two or more signals. However, they do not have an integrative model that simultaneously explains multiple forms of signals introduced by an SV and are geared towards identifying discrete SV genotypes in normal diploid genomes. CloneHD [26] utilized both SNVs and CNVs in a hierarchical model for probabilistic inference, but it did not utilize other types of SVs. To our best knowledge, no method has been proposed to estimate continuous VAF for SVs in heterogeneous tumor samples.

We present in this paper, a maximum-likelihood method and a software tool called BreakDown that aim to address the aforementioned challenges in estimating VAF for SV in a heterogeneous tumor sample. Our method analytically integrates 3 forms of SV signals: read depth, discordant read pairing and end-clipping and thus considers more evidences than any existing tools. Our formulation expands beyond existing work that assumes monoclonality and allows us to include any SVs in clonal heterogeneity inference, an improvement over previous investigations that involved only SNVs (e.g., Pyclone [8], Sciclone [27] and ExPANDs [28]) or large CNVs. As a stand-alone tool, BreakDown can be applied in a NGS data analysis pipeline to enhance the accuracy and estimate VAFs for SVs nominated by any other discovery tools. We assessed the performance of BreakDown using both simulated and real cancer genome sequencing data and found that it can produce consistently satisfactory results. BreakDown was designed to be self-adaptive to different conditions (e.g., coverage, read length, etc.), normalized against biases (e.g., GC content), and robust to outliers. It builds in a scoring system that is calibrated with validation data and can accurately inform true error probabilities.

## Results

### A maximum likelihood VAF estimator

**Figure 1:**
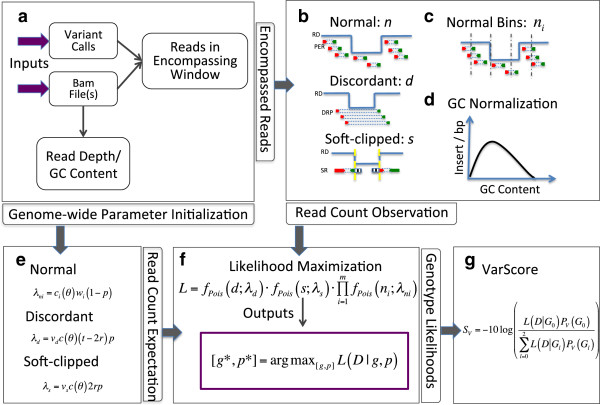
**Schematic overview of BreakDown. a)** In the input are the bam files and a set of SV calls. Reads encompassing each SV are extracted for analysis. Genome-wide parameters such as average read count per bp per GC content are initialized. **b)** The encompassed reads are divided into three groups: normal, discordant and soft-clipped. Reads in each group are counted. **c)** Normal reads for large CNVs are counted in a series of consecutive non-overlapping bins. **d)** Read counts are normalized by GC contents. **e)** Expected read counts are defined as functions of SV, VAF and sequencing data. **f)** Genotype and VAF that maximize the likelihood function are derived from the expected and observed read counts. **g)** Variant scores are estimated that quantify the confidence of the results. Detailed explanations of the mathematics are available in Methods.

We have developed an approach using maximum likelihood, which estimates the VAF of an SV that best explains the associated alignment data. Briefly, our approach includes the following five steps (Figure 1, see Methods for details). First, parameters such as average coverage and insert size are initialized based on measures from randomly selected regions of the genome. Read pairs encompassing an SV are extracted from the data (usually in BAM format [29]) (Figure 1a). Second, these read pairs are classified into three groups: A) normal, B) discordant and C) soft-clipped, based on their alignment patterns. The numbers of read pairs *n*, *d* and *s* are counted respectively in these 3 groups (Figure 1b-c). These counts are normalized with respect to (w.r.t.) GC content and mapping quality (Figure 1d). Third, given the expected numbers of counts (Figure 1e) and the observed counts *n*, *d* and *s*, the maximum likelihood estimation of VAF and genotype can be obtained. VAF is a continuous variable ranging between 0 and 1 and is suitable to represent allelic structure in heterogeneous samples, while genotype is a discrete variable (e.g., AA, AB, and BB) that is suitable in homogeneous samples. We keep both variables in one formulation to achieve unified maximal likelihood inference in either heterogeneous or homogeneous samples (Figure 1f). In this paper, we focus on measuring VAFs in heterogeneous tumor samples. A Bayesian variant score is computed to quantify the confidence of the results (Figure 1g).

Based on the above method, we implemented a software tool called BreakDown, which can be used in conjunction with SV discovery tools such as BreakDancer, GenomeSTRiP, Delly, CREST, Pindel, etc. to measure the genotype or VAF of candidate SVs in a BAM file.

### Characterizing the estimation accuracy

Our method involves sophisticated numerical calculations. Does it always return the correct results? To answer this question, we simulate read counts under various combinations of parameters including VAFs, variant types, variant sizes, sequence coverage, and insert sizes (Methods). We then ask BreakDown to estimate VAFs from simulated counts. To measure accuracy, we compute the chance of an estimated VAF falling near (±10%) the true value in 1000 random trials.

**Figure 2:**
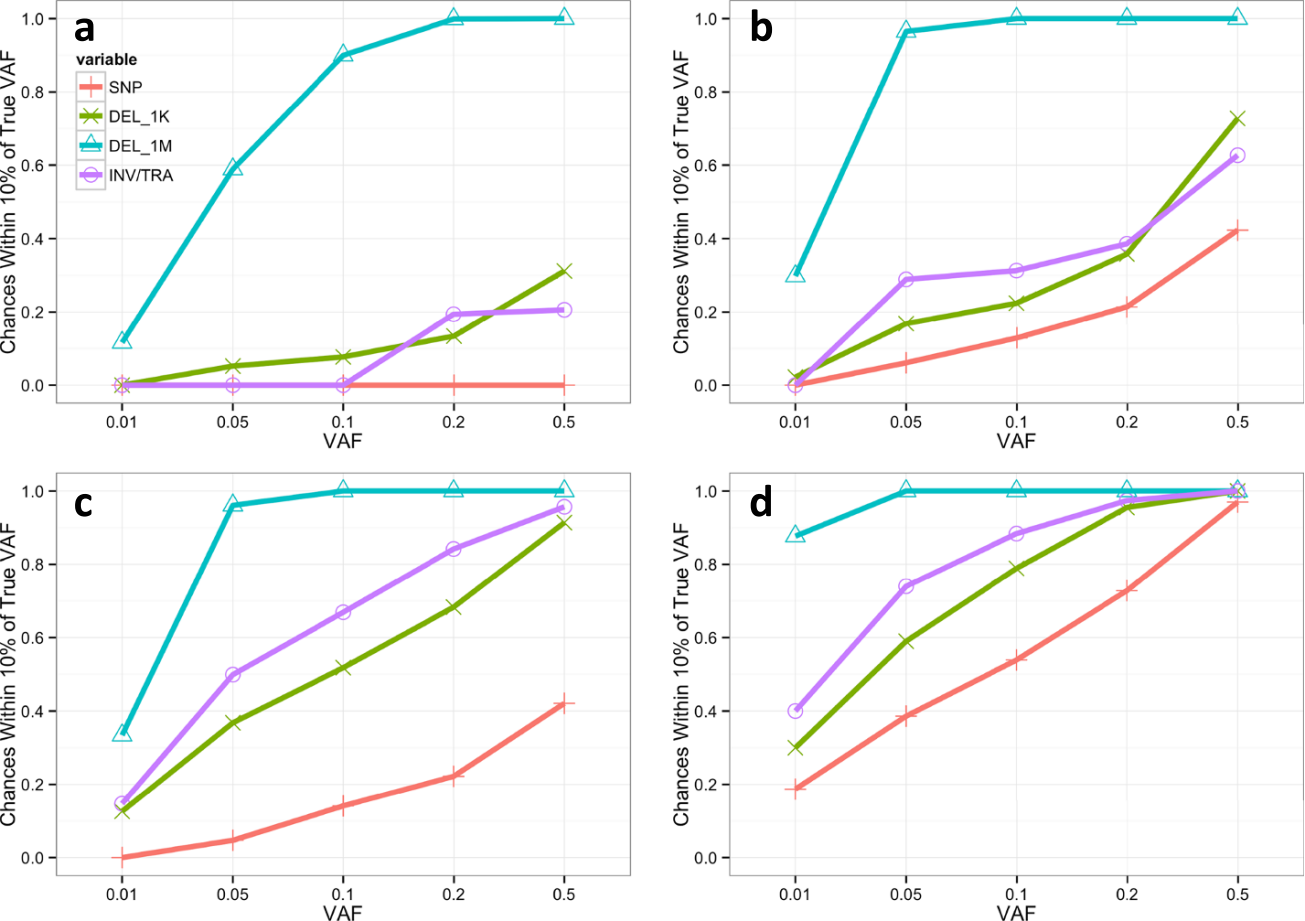
**Accuracy of estimated VAF in simulation.** Plotted are the chances (Y axis) of the estimated VAF falling within 10% of the true VAF (X axis). Each data point is estimated from 1000 random samples. Each subplot in the figure contains 4 curves representing the accuracy of VAFs estimated from SNVs (red plus), 1 Kb deletions (green cross), 1 Mb deletions (blue triangle), and inversions/reciprocal-translocations (purple circle). Various types of sequencing data are simulated and results compared: **a)** short-insert (500 bp) short-read (100 bp) at 5× sequence coverage, **b)** short insert short read at 30× coverage, **c)** long insert (3000 bp) short read at 30x coverage and **d)** short insert short read at 500× coverage.

From short-insert (500 bp) short-read (100 bp) low-coverage (5×) data (Figure 2a), it is very challenging to estimate VAF accurately from SNPs, medium-size deletions (1 Kb), inversions (INV) and reciprocal translocations (TRA). However, it is possible to accurately (≥90% chance) estimate relatively high (≥0.1) VAF from large (1 Mb) deletions. When coverage increases to 30X (Figure 2b), which is typically for WGS data, VAF as low as 0.05 can be accurately estimated from large deletions. Notably, VAF estimated from INVs and TRAs are more accurate than those estimated from SNVs, thanks to larger physical coverage than sequence coverage. Medium-size deletions perform always better than SNVs but worse than INVs and TRAs in low (<0.2) VAFs. However it outperforms INVs and TRAs at high (>0.3) VAFs. The accuracy of SVs over SNVs becomes even more striking as the insert size becomes longer (3 Kb) (Figure 2c), which indicates that our method has successfully leveraged physical coverage. Even at ultra-high (500×) coverage (Figure 2d), the SNVs still have limited accuracy (<0.6) in estimating small (<0.1) VAFs from short insert data. This result indicates that current methods that measure VAFs from only SNVs are suffering from great challenges in delineating low-abundance sub-clones, whereas when SVs are included, low-abundance sub-clones would have much higher chance to be identified.

In summary, this simulation results indicate that our method can accurately estimate VAFs for various types of SVs and can enhance the heterogeneity analysis from either short or long insert data at any coverage.

### Comparison with published tools

**Figure 3:**
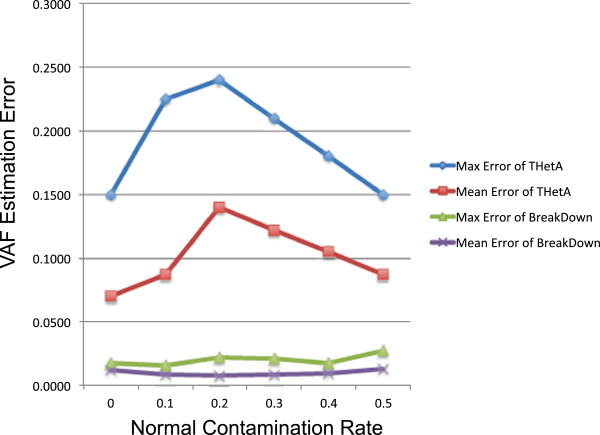
**Comparison of VAF estimation errors between BreakDown and THetA.** Plotted are the mean and the maximum errors (Y axis) estimated from 10 lineage specific SVs by BreakDown and THetA under six normal contamination rates from 0 to 0.5 (X axis).

The genomes of a tumor often evolve from a complex history that spans multiple years. To understand if BreakDown is more accurate than other tools in inferring complex history, we created a 5-clone mixture tumor sample, based on a mock phylogeny tree (see Methods, and Additional file 1: Figure S5). Each branch in the tree represents the birth of a new clone that contains two novel SVs. We generated synthetic reads from this bulk tumor genome. We also generated additional reads from the wild-type genome to simulate “normal contamination” that are frequently observed in real tumor samples. We created 6 data sets by varying the “normal contamination rate” from 0 to 0.5 with an incremental of 0.1. We applied both BreakDown and THetA on these 6 data sets and compared the accuracy of estimated VAFs (Methods). In all 6 sets, BreakDown achieved lower VAF estimation errors than THetA (Figure 3, Additional file 2: Table S1). We noticed that the accuracy of THetA starts to deteriorate when the normal contamination rate increases, whereas BreakDown achieved consistently small errors (<0.028). This result indicated that BreakDown is likely more accurate in modeling real tumor sample that often contain multiple tumor clones with variable rates of normal contamination.

### Identify somatic SVs from tumor-normal matched WGS data

To assess BreakDown’s capability in accurately estimating VAF and detecting somatic events in real data, we download the WGS data of a metastatic melanoma cancer cell-line COLO-829 and its matched normal cell-line [30]. For each putative SV, we compute its VAF in the tumor and the normal samples independently. We first run BreakDown on 32 previously validated deletions in the tumor [21, 30]. Except for 5 events, which are suspected to be germline (3 out of these 5 are also reported as germline by CREST), the estimated VAFs in the normal sample for the other 27 events (see Additional file 3: Table S2) have a mean of 0.024 and a standard deviation of 0.032, as expected for somatic deletions.

In the tumor sample, we observe that those events on chromosome 2, 3, 7, 17, 18, 20, 22 and X have VAFs diverging from 0, 0.5 or 1, which are unexpected from a homogeneous diploid sample. This implies aneuploidy on these chromosomes, assuming the tumor sample is pure. This speculation is confirmed by a previous independent study that characterizes the genome-wide copy number profile in this sample [31]. On chromosomes 5, 10, 15 and 16 that are indicated as mostly diploid [31], the estimated VAFs are within 0.06 of either 0.5 or 1.0 (see Additional file 1: Figure S3). Thus, the VAFs estimated by Breakdown in both the tumor and the normal samples appear to be valid.

**Figure 4:**
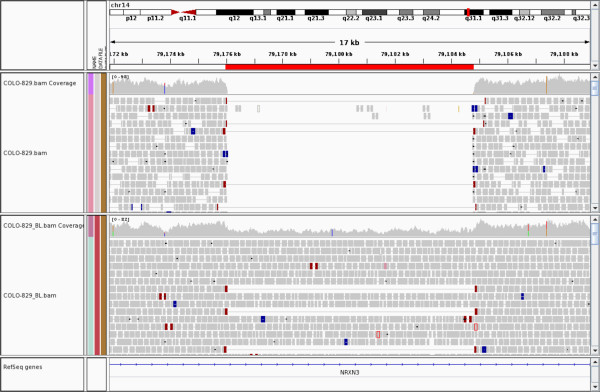
**A loss-of-heterozygosity deletion in** ***NRXN3*** **in COLO-829.** Plotted is an image from the integrative genome viewer (IGV) that represents read alignment over this deletion (chr14:79175898–79184805) in the tumor sample (top panel) and in the normal sample (bottom panel). The discordant read pairs (dark-red bars connected by long grey lines) and drop in coverage (white space in the center) correspond to the start and the end of the deletion (red horizontal bar). VAFs are estimated by BreakDown to be 1.0 and 0.49, respectively in the tumor and the normal.

We run BreakDancer on the tumor and the normal samples independently, and run Breakdown on the resulting deletion calls. Since previous studies have been fairly comprehensive at identifying somatic heterozygous deletions (gain-of-heterozygosity or GOH) we focus on identifying loss-of-heterozygosity (LOH) events. We find 41 candidate LOH events that have VAFs between 0.45 and 0.55 in the normal and between 0 and 0.05 or 0.95 and 1 in the tumor (see Additional file 4: Table S3). Among them, 40 (97.6%) overlap previously reported LOH regions deriving from segmenting the B-allele frequency of SNPs [30]. Two LOHs are of potential functional impact. One at chr10q22.3 hits *C10orf11*, a melanocyte-differentiation gene that has been related with autosomal-recessive albinism in humans [32]. This homozygous deletion is found overlapping the only homozygous region found in four affected individuals but not in any unaffected ones. The other LOH at chr14q31.1 hits gene *NRXN3* (Figure 4), which has been related to malignant melanoma [33].

### Identify sub-clonal SVs in breast cancer samples

We analyzed three pairs of matched tumor-normal breast cancer samples to show that BreakDown can accurately estimate VAFs of medium-size deletions, inversions and translocations. The first pair consists of an estrogen-receptor (ER)-positive primary breast cancer sample PD4120, sequenced by Illumina Hi-seq at 188×, and a matched normal sample at 38x. The clonal structure of this tumor has been previously inferred based on SNVs and large CNVs [2, 19, 26]. The other two tumor samples PD4115 and PD4088 were sequenced at around 40× and their clonal structure have been characterized by THetA [19]. For these 3 cases, we called structural variation using BreakDancer on the paired tumor and normal samples. We then run BreakDown on each of the candidate SV calls. Through this process we discovered several subclonal deletions (all of which are shorter than 10 Kb), inversion and translocation (Table 1) that have not been previously reported [34].

**Table 1:** **Novel sub-clonal somatic structural variants detected by BreakDown**

SAMPLE	CHR1	POS1	CHR2	POS2	TYPE	Size(bp)	GENE	EST_VAF
**PD4120**	10	117351105	10	117352856	Deletion	1751	*ATRNL1*	0.12
**PD4120**	5	86223184	5	86225988	Deletion	2804		0.38
**PD4120**	14	51889637	14	51896099	Deletion	6462		0.39
**PD4120**	22	19372768	22	21122617	Translocation	N/A	*HIRA, PI4KA*	0.055
**PD4115**	1	10151402	1	10152851	Deletion	1449	*UBE4B*	0.296
**PD4115**	9	108351356	9	108352767	Deletion	1411	*FKTN*	0.285
**PD4115**	9	140773612	9	140777195	Deletion	3583	*CACNA1B*	0.400
**PD4115**	X	73061886	X	73067477	Deletion	5591	*XIST*	0.394
**PD4088**	10	60908547	10	60920370	Inversion	11823		0.594

Listed are the novel somatic structural variants in the breast cancer sample PD4120, PD4115 and PD4088 along with BreakDown estimated VAFs (column 9). Also shown (column 8) are the genes overlapping with either deletion loci or translocation breakpoints.

**Figure 5:**
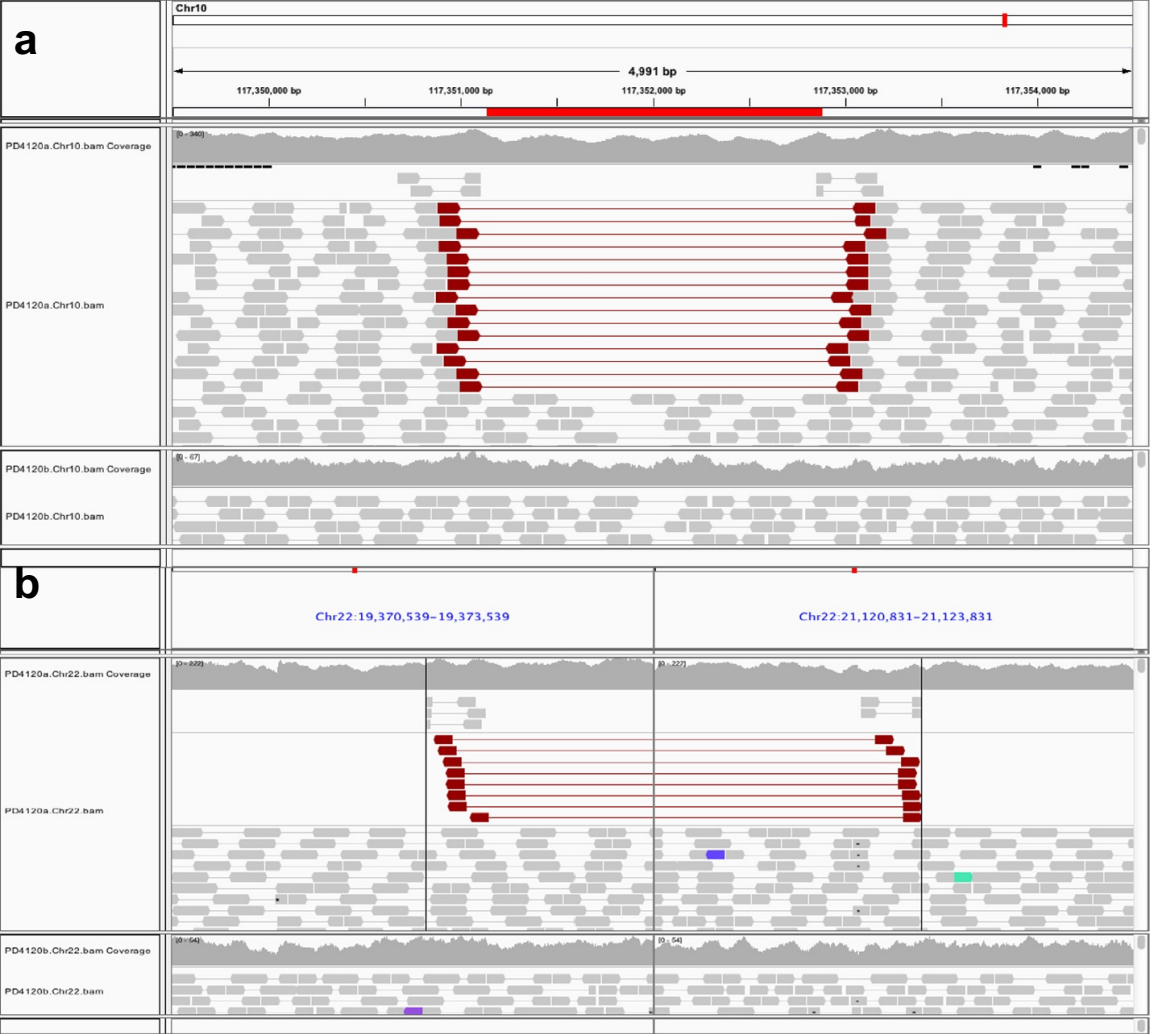
**Sub-clonal SVs in PD4120.** Plotted are the integrative genome viewer (IGV) screenshots that display reads in the tumor sample (top panel) and in the normal sample (bottom panel) of **a)** A 1751 bp deletion between Chr10:117351105 and Chr10:117352856 with an estimated tumor VAF 0.12, and **b)** an intra-chromosomal translocation between Chr22:19372768 and Chr22:21122617 with an estimated tumor VAF 0.055. Reads are displayed from the top to the bottom in the following order: split reads (partially clipped bars), discordant read pairs (brown bars and lines), and normal read pairs.

For the novel somatic SVs detected in PD4120, two deletions (at chr5q14.3 and chr14q22.1) have BreakDown predicted VAFs of 0.38 and 0.39, respectively (see Additional file 1: Figure S4A and b). Because the tumor purity of PD4120 is around 70% [2, 19], these two deletions are thereby likely heterozygous deletions in the primary clone (cluster D in [2]). The third deletion (at chr10q25.3) (Figure 5a) has a predicted VAF of 0.12, which may originate from one of the sub-clones (cluster B in [2]) that has a SNV VAF peak at 0.11. It can be seen (Figure 5a) that although the read depth signal is not apparent, there are 15 discordant read pairs and 4 split reads, all of which are important for estimating VAF but have been ignored in the previous studies. The translocation (Figure 5b) has a BreakDown estimated VAF of 0.055, which matches another sub-clone (cluster A in [2]) with a SNV VAF peak at 0.05. For the four deletions discovered in PD4115 (76% tumor purity [19]), two (at chr9q34.3 and chrXq13.2) have VAFs estimated at around 0.4, which implies that they are likely heterozygous variants in the founding clone. The other two (at chr1p36.22 and chr9q31.2) had estimated VAFs at around 0.3, and are likely homozygous deletions in the subclone of 32.7% abundance [19]. One novel somatic inversion (at chr10q21.1) that we found in PD4088 (59% tumor purity [19]) has an estimated VAF 0.594, which may be a homozygous event in the founding clone. In summary, all of the novel somatic SVs we have identified were consistent with previously inferred clonal architecture, which demonstrated the accuracy of our method.

## Discussion

In this paper, we presented a novel sequence analysis method that can estimate the VAFs of SVs from a heterogeneous tumor sample.

We showed that VAFs estimated from SVs by our methods are at least several times more accurate than those estimated from SNVs, thanks to our integration of diverse alignment signals (or coverage) from multiple groups of reads. We also clearly demonstrated that different types of SVs are associated with different accuracy in VAF estimation and our method can fully harness the structural nature of these SVs.

Our work has extended current clonal inference from SNVs and large CNVs to include medium-size SVs and balanced SVs such as inversions and translocations. It is possible to further extend our model to account for complex SVs such as chromothripsis [35–37] and chromoplexy [38, 39].

In terms of accurately estimating VAFs, our approach compared favorably to existing tools. In our simulation, our model could more reliably estimate VAFs than THetA from tumor samples that have multiple clones and a high level of normal contamination. Other approaches such as ABSOLUTE were not directly comparable to our approach, because they were designed to infer tumor purity and ploidy without further characterizing clonal structure or subclonal mutations [7].

In our analysis of COLO-829, we identified 40 putative germline deletions in somatic LOH regions. These events, although potentially important, have not been systematically reported in previous studies that examined the same set of data. Therefore, the results obtained by our approach can facilitate more accurate characterization than the previous studies that utilized only SNVs and indels [30]. Our finding also indicated that previous studies might have narrowly focused on detecting GOHs but somewhat ignored LOHs. As medical genomics research continues, we expect that our method will have a tremendous opportunity to improve the comprehensiveness of mutational profiling from an unprecedented amount of WGS data that are accumulated by the tumor genome atlas, the international cancer genome consortium [40] and broader biomedical research communities.

The analysis of breast cancer samples PD4120, PD4115 and PD4088 demonstrated the identification of subclone SVs using BreakDown. The estimated VAF, together with those from SNVs and large CNVs, will serve as input to further infer clonality, ploidy and purity. While these three perspectives are intermingled, we believe that incorporating SVs into the picture [7, 8, 19, 26] will greatly enhance the quality of the inference.

Besides heterogeneous tumor samples, our method can also be applied to infer the genotypes of normal homogeneous WGS samples such as those in the 1000 genomes projects [41]. We will report our findings in a separate manuscript.

## Conclusions

We have developed a maximal likelihood framework, which integrates multiple forms of alignment signals to estimate the allele fraction of a structural variant. Our methods and software tool BreakDown can estimate the allele fraction of most structural variants including small and large deletions, balanced inversions and translocations. Evaluation based on both simulated and real data indicates that our method outperforms existing approaches and can greatly enhance the characterization of intra-tumoral heterogeneity in genomically instable tumors.

## Methods

### The Maximum Likelihood formulation

Our method starts from a NGS paired-end BAM file produced by BWA [42] and a set of SV calls produced by BreakDancer, Delly, Pindel and other SV discovery tools. All these tools predict a start and an end coordinates and the variant types such as deletion, duplication, translocation and so on (Figure 1a).

The VAF or the genotype of a variant is determined by maximizing the following likelihood function:


where *D* denotes the alignment data in a window *w* that encompasses the SV, and *p* the variant allele fraction (VAF) ranging from 0 to 1. In a monoclonal diploid genome, we use *g* to represent genotypes (AA, AB, BB), which are equivalent to *p* equals to 0, 0.5, and 1, respectively. Including both *p* and *g* makes it convenient to apply our method to both heterogeneous and homogeneous samples. The analytic form of the likelihood function *L* is parameterized by read length *r*, insert size *t*, average number of inserts (read pairs) per bp *c*, which are estimated from normal diploid regions of the genome.

BreakDown classifies read pairs into three groups based on their alignment to the reference and counts the numbers of: 1) normal read pair *n*, 2) discordant read pair (DRP) *d*, and 3) soft-clipped reads (SR) *s* in *w* (Figure 1b). The definitions of these read groups are similar to previous work [17, 43]. Soft-clipped reads are recognized from the CIGAR strings in the BWA alignment (Figure 1b, [44]). We define a read pair as normal if its two reads align with reference in expected orientation and distance, or otherwise as discordant.

An SV typically associates with three counts D = {*n*,*d*,*s*}. However, for a balanced SV such as an inversion or a reciprocal translocation, the normal counts are irrelevant: D = {*d*,*s*}. If an SV contains multiple breakpoints, *d* and *s* each becomes an array of counts from constituent breakpoints.

Unlike other SV detection methods, which sequentially analyze these different groups of reads [23–25], we jointly analyze all the reads in *w*. Because read pairs in these 3 groups are sequenced and aligned independently, the likelihood function can be expanded into the following product:


which probabilistically integrates different types of counts. In an unbiased shotgun sequencing experiment, these counts should follow Poisson distributions with parameter *λ* being defined in the following sections [45].

### Modeling normal read pairs

An SV such as a copy number variant (CNV) can span a very large genomic region with excessive GC content variation. It is known that GC content can introduce substantial sequencing bias and need to be normalized against (see Additional file 1: Figure S1) [46]. In our method, instead of having one normal count *n* from the entire length of the encompassing window *w*, we split *w* into smaller non-overlapping bins *w*_*i*_ and count in each bin the number of normal read pairs *n*_*i*_. We assume that *n*_*i*_ follows a Poisson distribution with


where *c*_*i*_ is the average number of normal read pairs per bp, normalized by the GC content in *w*_*i*_. We can pre-estimate *c*_*i*_(*θ*) as a function of GC-content *θ* from randomly selected regions in the genome (Figure 1d). *w*_*i*_ has a fixed width of 100 bp under our default setting. Splitting a large window into small bins allows normalization being performed at fine resolution and is particularly effective for large SVs that span GC-content variable region. As another corrective measure, we exclude bins that contain more than 50% of zero mapping quality reads, an indicator of potentially unreliable data that are introduced by mapping errors in repetitive regions.

### Modeling discordant read pairs

The expected count of DRP *d* should be linear to the span coverage [47], i.e., insert size *t* minus twice read length *r* (Figure 1b):


where *c*(*θ*) denotes the mean number of inserts per bp, a function of the GC-content. The observed numbers of DRPs often turn to be smaller than what is expected from the span coverage due to simplification in the above definition and peculiarities of alignment algorithms. We used *v*_*d*_, a trainable parameter ranging between 0 and 1 to compensate such offset. In our experience, *v*_*d*_ is around 0.8 in typical WGS data (see Additional file 1: Figure S2).

### Modeling soft-clipped read

The expected number of soft-clipped read *s* should be proportional to the summation of read length (sequence coverage) in a read pair (Figure 1b), assuming a read would become soft-clipped if it has any overlap with the breakpoint:


This formulation is approximate because an aligner may choose not to soft-clip a read when it only slightly overlaps the breakpoint. Sometimes, an aligner may incorrectly soft-clip a non-breakpoint containing read. However, such aligner-specific behaviors can hardly be modeled post-alignment. To alleviate this bias, we use a trainable parameter *v*_*s*_ to compensate for such offset. In our experience, *v*_*s*_ is around 0.7 in typical WGS data (Additional file 1: Figure S2).

### VAF estimation

Taken together, we can now express the likelihood function as


where *m* denotes the number of bins for counting normal read pairs (Figure 1c). For genomes sequenced with multiple DNA libraries, the quantities estimated from each library are combined through multiplication, assuming that the libraries are independently constructed. Without loss of generalizability, we present the derivation of VAF from a single library.

Solving equation *dL*/*dp* = 0 yields the variant allele fraction that maximizes the likelihood function in a close-form quadratic solution,


where


### Confidence scoring

We use variant score (VarScore) to quantify error probability, i.e., the chance that there is no SV at the input site (Figure 1g):


where *P*(*p* = 0|*D*) represents the posterior probability that VAF equals to 0 given the data. For practical implementation, we used discretized genotype to estimate the error probabilities:


where *P*(*g* = *AA*|*D*) is the posterior probability that the genotype is homozygous reference. We can calculate this quantity based on Bayesian Theorem:


where *P*_*V*_(*G*) is the prior variant probability of genotype *G* and *G*_*l*_, *l* = 0, 1, or 2 represents homozygous reference, heterozygous variant and homozygous variant genotypes, respectively. For a heterogeneous tumor sample, uniform genotype prior is assumed *P*_*V*_(*G*) =1. For a homogeneous normal sample, the genotype prior can be defined based on population genetics [23]. Assuming Hardy-Weinberg equilibrium,


where *q* is the average allele frequency of the SVs in the population.

### Genome-wide parameter initialization

Parameters that are needed by our model are initialized from the data before they are applied to VAF estimation. We randomly choose *N* (*N*=10 by default) 10 Mb regions from the BAM file (excluding centromere and telomere regions). We estimate median read length *r*, insert size *t* from the data. We create a lookup table that stores average read pair per bp *c*_*i*_(*θ*) as a function of GC content (an integer ranging from 0 to 100) (see Additional file 1: Figure S1).

### Simulation

To examine the accuracy and robustness of our maximum likelihood estimators and to characterize different parameters, we simulated a set of read counts for SNPs, deletions (with size 1K and 1M bp), inversions or reciprocal translocations at coverages of 5X, 30X and 500X based on short insert size (500 bp) and short read length (100 bp). We also simulated read counts from long insert size (3000 bp) and short read length (100 bp) at 30X coverage (Figure 2c).

For each parameterization, we randomly sampled 1000 data points from the Poisson distributions (as described previously). For SNVs, we assumed that the number of variant supporting reads follows a binomial distribution parameterized by the given coverage and VAF. For an inversion or a reciprocal translocation that have two breakpoints, counts at each breakpoint were simulated independently.

### Comparison with THetA

We simulated five alterative copies of chromosome 20 (chr20), each containing unique SVs, as represented on the leaf nodes of a phylogeny tree (see Additional file 1: Figure S5). Each of the five clones contains two or four randomly placed non-overlapping 1.5 Mb heterozygous deletions or one-copy tandem duplications. Each clone makes up to a fraction of the total tumor mass. We used wgsim to simulate reads from each chr20 sequences. The corresponding coverages are calculated according to their clonal fraction and the normal contamination rate, which equaled to 0, 0.1, 0.2, 0.3, 0.4 or 0.5 in our simulation. The total coverage was kept at a constant 50X across all conditions. All the deletions and the duplications were simulated as single copy alterations, and therefore the true VAF ranged from 0.05 to 0.3 when the normal contamination rate is 0. When the normal contamination rate is 0.5, the true VAFs ranged from 0.025 to 0.15. We mapped the synthetic reads to the wide-type chr20 reference using bwa-mem [42].

We ran THetA beta version 0.60 under default parameters. The whole chr20 was segmented into 19 regions, corresponding to 10 non-overlapping copy number alterations with copy number neutral regions in between. The interval count file, serving as input to THetA, was generated by counting reads aligned into each of the 19 regions, for both the tumor and the normal samples. This version of THetA supported the inference of up to 3 clones. However, it reported that n=3 was not a good model for this data. Therefore, all the results we reported from THetA are based on n=2, i.e., one tumor clone plus one contaminating normal clone. Since the maximum copy number THetA estimated was 3, we converted copy number of those 10 intervals into VAF by , in which *C*_*i*_ represents the copy number estimated for the *i* th interval, and *μ* the estimated normal contamination rate.

### Data

COLO-829 NGS data was downloaded from the European Genome-Phenome Archive (Accession number: EGAD00000000055). CREST and validated call set was from Additional file 3: Table S2 (nmeth.1628-S2) downloaded from [21]. The LOH set was obtained from the Supplementary Table six from [30].

The NGS data for the breast cancer samples were downloaded from the European Genome-Phenome Archive (Accession number: EGAD00001000138). Validated SV set was obtained from Supplementary Table one from [34].

### Software availability

The BreakDown source code and manual are available for download at [48].

## Electronic supplementary material

Additional file 1: Figure S1: GC content biases in read counts. **Figure S2.** Comparison between the observed and the expected read counts. **Figure S3.** Plots of estimated VAF of validated deletions. **Figure S4.** Plots of two novel somatic deletions identified from the breast cancer sample. **Figure S5.** A mock phylogeny tree of a polyclonal tumor mass. (PDF 520 KB)

Additional file 2: Table S1: Comparison of the VAF estimation errors between BreakDown and THetA based on simulation. (XLSX 42 KB)

Additional file 3: Table S2: A list of 32 previously reported deletions with BreakDown estimated VAFs in COLO-829. (XLSX 52 KB)

Additional file 4: Table S3: A list of 41 LOH deletions detected by BreakDown in COLO-829. (XLSX 46 KB)

## Abbreviations

SV: Structural variation
NGS: Next generation sequencing
VAF: Variant allele fraction
CNV: Copy number variation
bp: Base pair
WGS: Whole genome sequencing.
