# Supplementary material for: Towards accurate characterization of clonal heterogeneity based on structural variation
Source: BMC Bioinformatics. 2014 Sep 8;15(1):299. doi: 10.1186/1471-2105-15-299 (PMC4165998; doi:10.1186/1471-2105-15-299)
Supplement: Supplementary file 1 — Additional file 1: Figure S1: GC content biases in read counts. Figure S2. Comparison between the observed and the expected read counts. Figure S3. Plots of estimated VAF of validated deletions. Figure S4. Plots of two novel somatic deletions identified from the breast cancer sample. Figure S5. A mock phylogeny tree of a polyclonal tumor mass. (PDF 520 KB) [file 12859_2014_6578_MOESM1_ESM.pdf]

Figure S1, GC content biases in read counts.

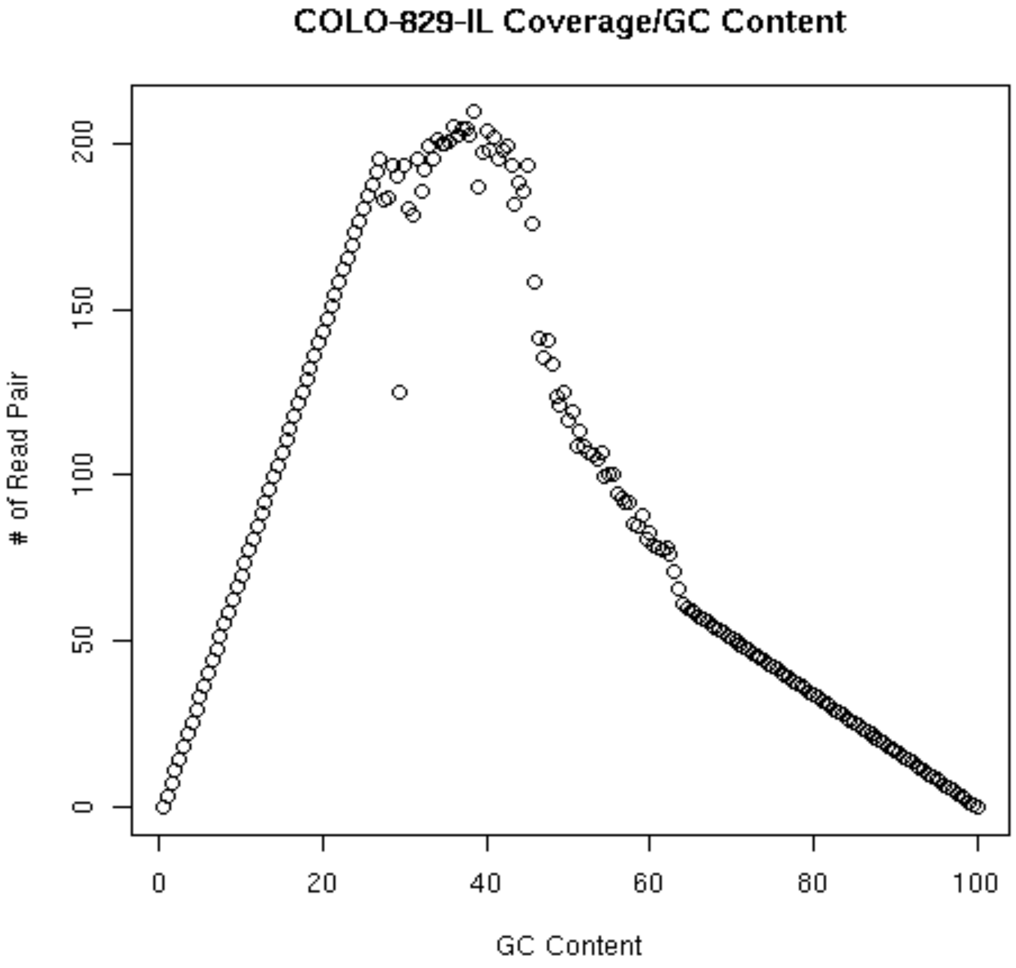


(a)

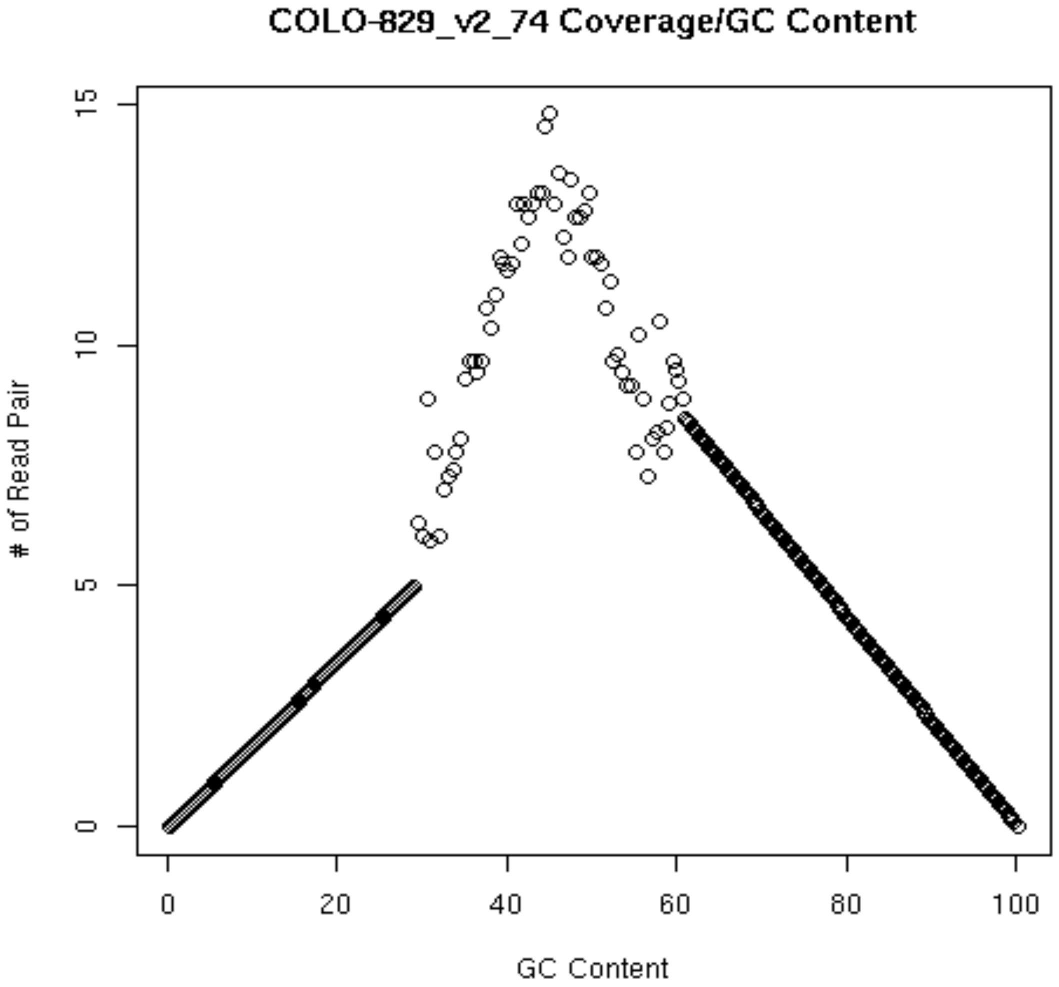


(b)

The number of read pairs is estimated by randomly selecting 10 regions of size 10Mbp in the whole genome, each region further divided into 10K non-overlapping small regions. The number of read pairs per bp and corresponding GC content are calculated from each 10K region. Plotted are the median numbers of read pairs per bp (Y axis) at different GC content (X axis) in two DNA libraries: (a) COLO-829-IL and (b) COLO-829_v2_74. The straight lines on the left and the right sides of the curve are linearly extrapolated from the real values in the middle.

Figure S2, Comparison between the observed and the expected read counts.

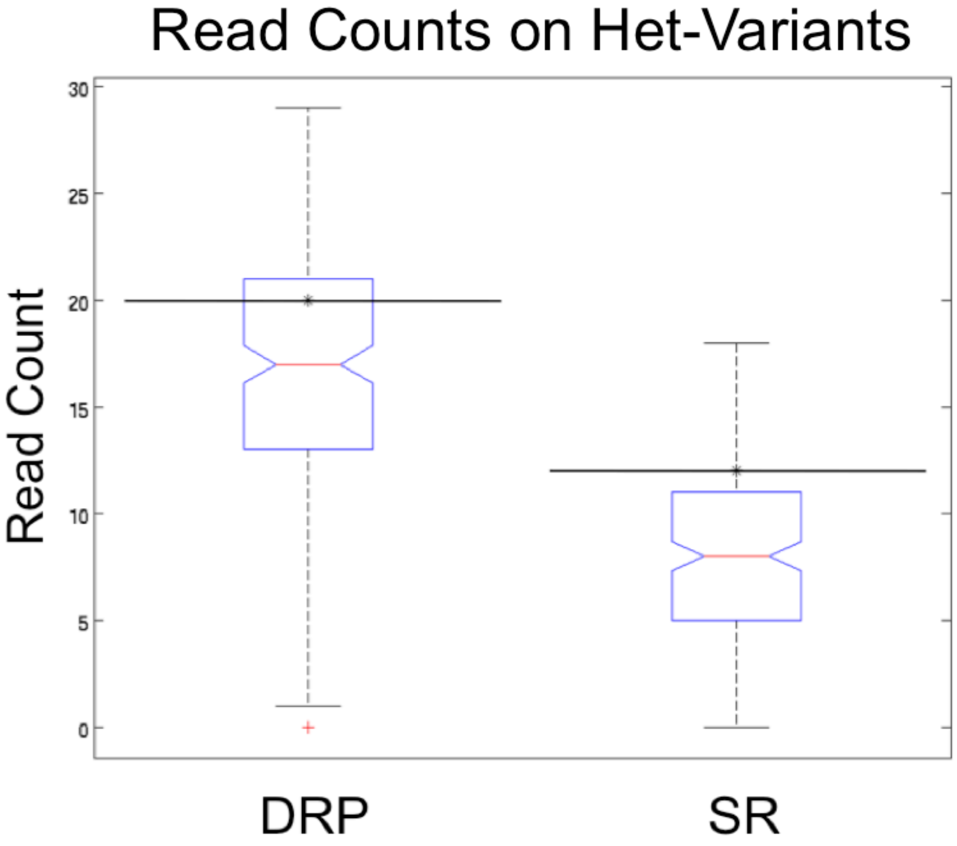


(a)

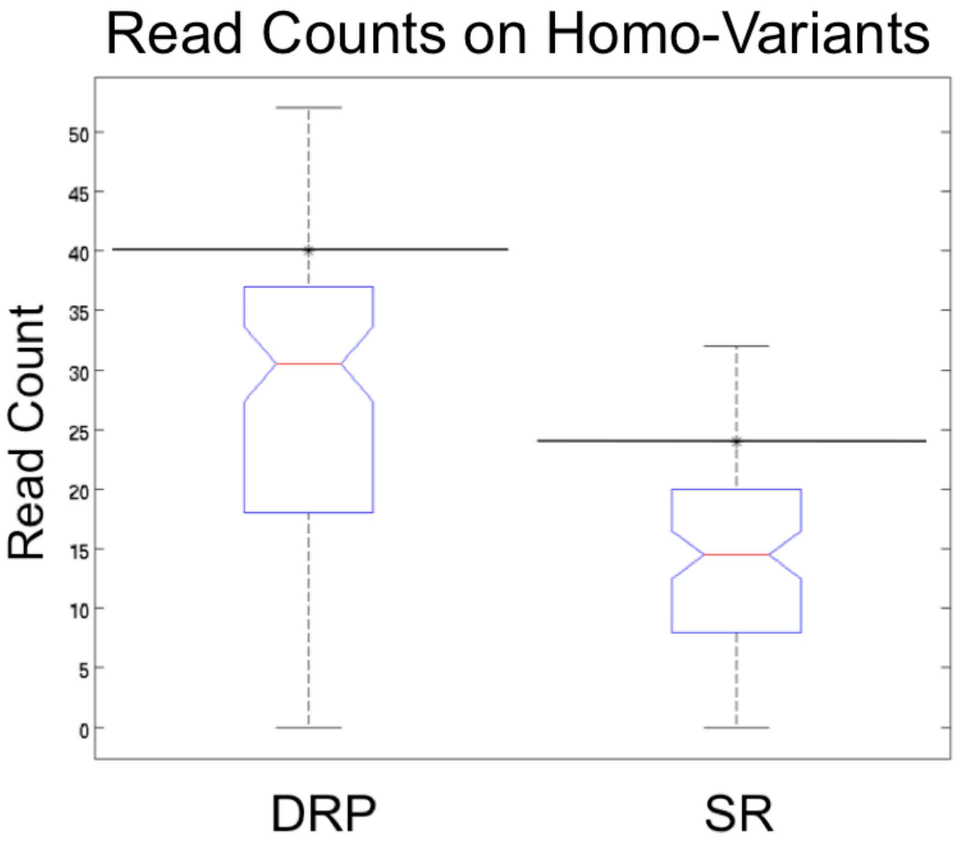


(b)

Data in boxplots are the number of discordant read pairs and soft-clipped reads from the known deletions from 1000 Genomes Project sample NA12878 [1] with 67x sequence coverage and 100 bp long reads. The boxes represent the 75% and 25% percentiles on the high and low ends. The red horizontal lines indicate the medium, i.e. 50% percentile. The highest and lowest horizontal lines outside of the box indicate the maximum and minimum counts. Expected read counts for the heterozygous variants (a) and homozygous variants (b), corresponding to VAFs of 0.5 and 1, respectively are plotted as black horizontal lines above the boxes.

Figure S3, Plots of estimated VAF of validated deletions.

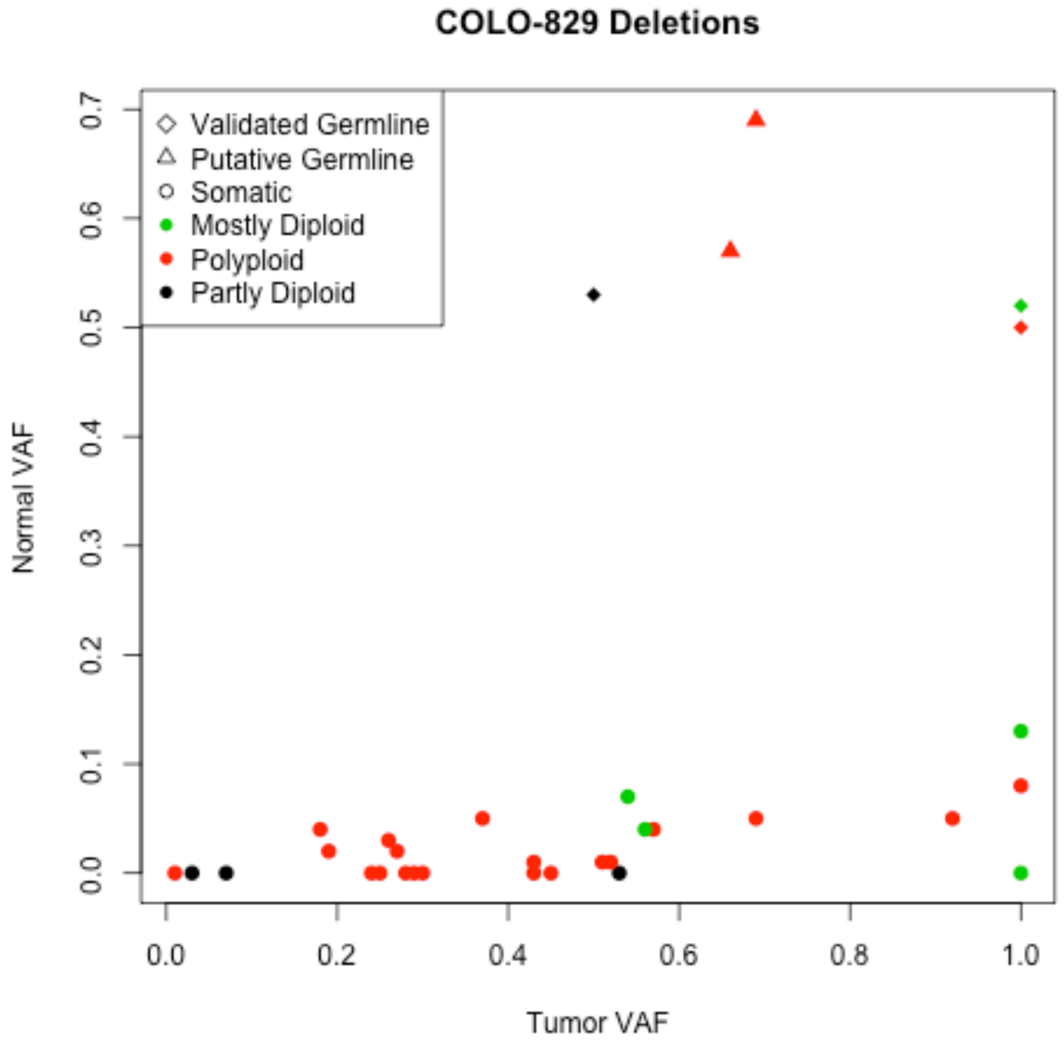


Diamond, triangle and circles represent validated germline, putative germline and somatic events. For tumor ploidy analysis, those events on the chromosomes which are mostly diploid are in green, and mostly polyploidy in red. Those that are partly diploid are in black.

Figure S4, Plots of two novel somatic deletions identified from the breast cancer sample PD4120.

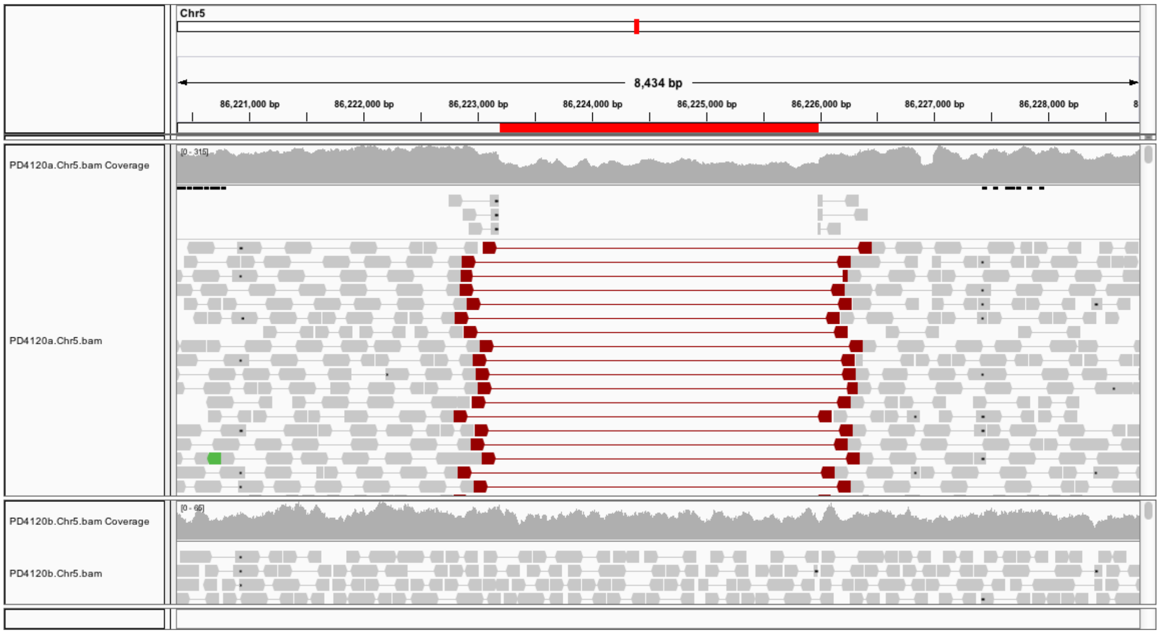


(a)

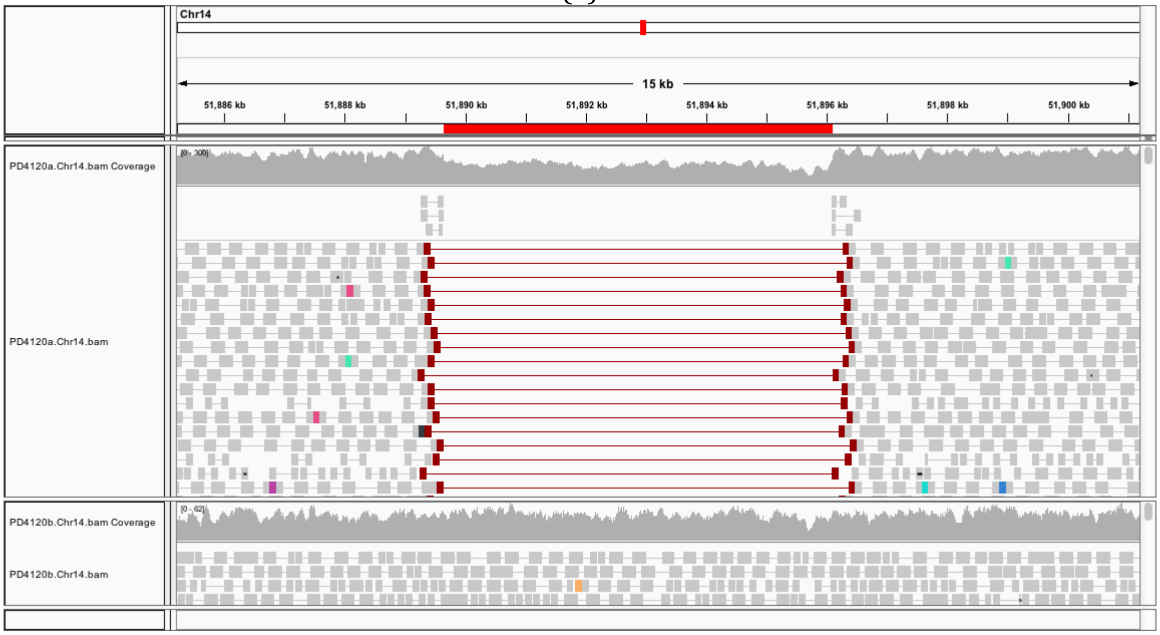


(b)

Displayed in each plots are the read alignments in the tumor sample (top panel) and in the matched normal sample (bottom panel). The red horizontal bar on the top labels the deleted regions. In each panel from top to bottom are split reads (partially clipped bars), discordant read pairs (brown bars and lines), and normal read pairs. (a) The clonal deletion between Chr5:86223184 and Chr5:86225988 with an estimated tumor VAF 0.38. b) The clonal deletion between Chr14:51889637 and Chr14:51896099 with an estimated tumor VAF 0.39.

Figure S5. A mock phylogeny tree of a polyclonal tumor mass.

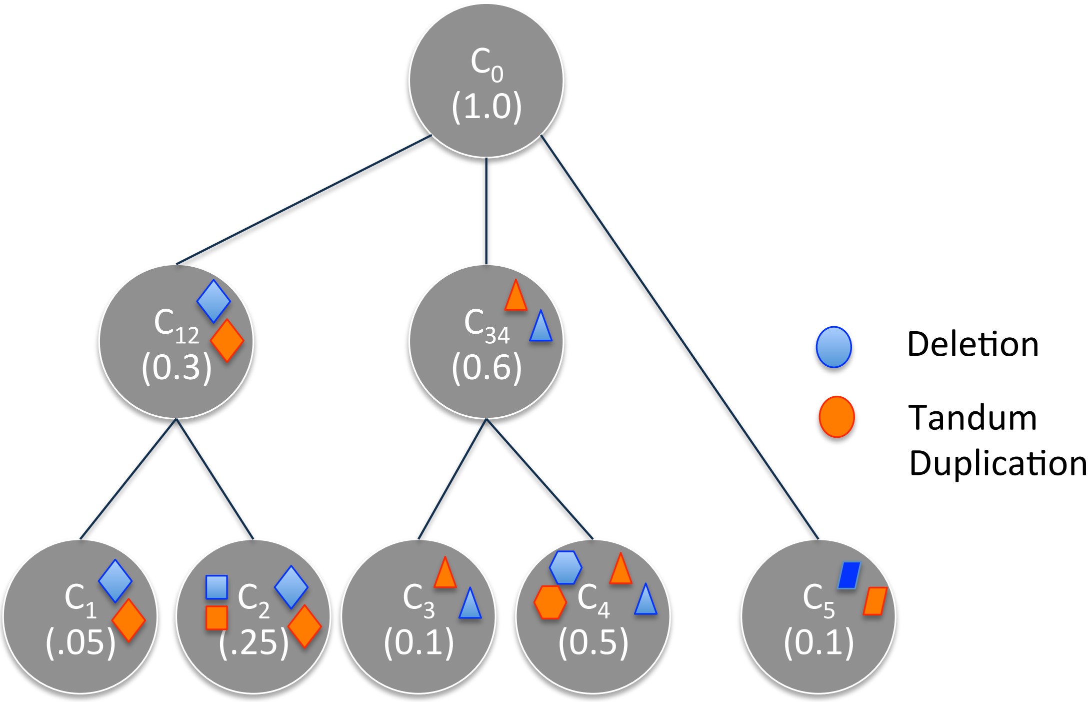


The root is $C_0$, representing the founding germline clone. Each node, $C_0$ to $C_5$, corresponds to a clone that existed in the phylogeny. $C_1$ and $C_2$ evolved from $C_{12}$, $C_3$ and $C_4$ evolved from $C_{34}$ and $C_5$ evolved directly from $C_0$. The ratios in the parentheses (ranging from 0.05 to 1.0), represents the relative abundance of a clone at the time of branching. The SVs in different clones are labeled in different shapes based on their lineages. SV types are differentiated by colors: blue for deletion, orange for tandem duplication. To simulate progressive tumor clone development, both $C_1$ and $C_2$ inherited the two SVs (diamond) in $C_{12}$, whereas $C_2$ had two novel SVs (square). The similar labeling was applied to $C_{34}$, $C_3$, $C_4$, and $C_5$.

**Reference**

[1] Conrad DF, Pinto D, Redon R, Feuk L, Gokcumen O, Zhang Y, Aerts J, Andrews TD, Barnes C, Campbell P, Fitzgerald T, Hu M, Ihm CH, Kristiansson K, MacArthur DG, MacDonald JR, Onyiah I, Pang AW, Robson S, Stirrups K, Valsesia A, Walter K, Wei J, Tyler-Smith C, Carter NP, Lee C, Scherer SW, Hurles ME: **Origins and functional impact of copy number variation in the human genome.** *Nature* 2009, **464**(7289)**:**704–712.
